# Combined DNA extraction and antibody elution from filter papers for the assessment of malaria transmission intensity in epidemiological studies

**DOI:** 10.1186/1475-2875-12-272

**Published:** 2013-08-02

**Authors:** Amrish Baidjoe, Will Stone, Ivo Ploemen, Shehu Shagari, Lynn Grignard, Victor Osoti, Euniah Makori, Jennifer Stevenson, Simon Kariuki, Colin Sutherland, Robert Sauerwein, Jonathan Cox, Chris Drakeley, Teun Bousema

**Affiliations:** 1 Department of Medical Microbiology, Radboud University Nijmegen Medical Centre, Nijmegen, the Netherlands; 2 Centre for Global Health Research, Kenya Medical Research Institute and Centers of Disease control and prevention, Kisumu, Kenya; 3 Department of Disease Control, Faculty of Infectious and Tropical Diseases, London School of Hygiene and Tropical Medicine, London WC1E 7HT, UK; 4 Department of Immunology & Infection, Faculty of Infectious and Tropical Diseases, London School of Hygiene and Tropical Medicine, London WC1E 7HT, UK

**Keywords:** *Plasmodium*, Malaria, Antibodies, IgG, DNA, Submicroscopic, Extraction, Transmission, PCR, ELISA

## Abstract

**Background:**

Informing and evaluating malaria control efforts relies on knowledge of local transmission dynamics. Serological and molecular tools have demonstrated great sensitivity to quantify transmission intensity in low endemic settings where the sensitivity of traditional methods is limited. Filter paper blood spots are commonly used a source of both DNA and antibodies. To enhance the operational practicability of malaria surveys, a method is presented for combined DNA extraction and antibody elution.

**Methods:**

Filter paper blood spots were collected as part of a large cross-sectional survey in the Kenyan highlands. DNA was extracted using a saponin/chelex method. The eluate of the first wash during the DNA extraction process was used for antibody detection and compared with previously validated antibody elution procedures. Antibody elution efficiency was assessed by total IgG ELISA for malaria antigens apical membrane antigen-1 (AMA-1) and merozoite-surface protein-1 (MSP-1_42_). The sensitivity of nested 18S rRNA and cytochrome b PCR assays and the impact of doubling filter paper material for PCR sensitivity were determined. The distribution of cell material and antibodies throughout filter paper blood spots were examined using luminescent and fluorescent reporter assays.

**Results:**

Antibody levels measured after the combined antibody/DNA extraction technique were strongly correlated to those measured after standard antibody elution (p < 0.0001). Antibody levels for both AMA-1 and MSP-1_42_ were generally slightly lower (11.3-21.4%) but age-seroprevalence patterns were indistinguishable. The proportion of parasite positive samples ranged from 12.9% to 19.2% in the different PCR assays. Despite strong agreement between outcomes of different PCR assays, none of the assays detected all parasite-positive individuals. For all assays doubling filter paper material for DNA extraction increased sensitivity. The concentration of cell and antibody material was not homogenously distributed throughout blood spots.

**Conclusion:**

Combined DNA extraction and antibody elution is an operationally attractive approach for high throughput assessment of cumulative malaria exposure and current infection prevalence in endemic settings. Estimates of antibody prevalence are unaffected by the combined extraction and elution procedure. The choice of target gene and the amount and source of filter paper material for DNA extraction can have a marked impact on PCR sensitivity.

## Background

To effectively implement and evaluate malaria control efforts a detailed knowledge is required of *Plasmodium* carriage and transmission within target populations. Transmission intensity is traditionally assessed using mosquito trapping techniques to determine exposure to infected *Anopheles* mosquitoes. In low endemic areas, where vector populations may be sparsely infected, small or heterogeneously distributed, trapping becomes operationally and technically unattractive [1-3]. A frequently used alternative is the prevalence of malaria infection in human populations, which is typically assessed by light microscopy. However, the limited detection limit and operational constraints of microscopical surveillance present a major barrier to its application in low endemic areas [4-8]. With patterns of reducing malaria transmission intensity in many African settings [9-14], it will become increasingly important to have sensitive alternatives for population level surveillance in areas approaching a phase of elimination [7,15].

Serological and molecular tools have been proposed to be particularly useful for monitoring transmission intensity and determining parasitaemia among populations in areas of low endemicity. Antibody responses to recombinant asexual malaria antigens are strongly associated with entomological measures of transmission intensity and microscopical parasite prevalence [16], but at low endemicity have a greater discriminative power [3]. Low level transmission may be detectable in the absence of microscopically detectable infection [17] and serological markers can detect spatial variation in transmission intensity [18] and the efficacy of interventions [19]. While serology can be used to detect spatial and temporal patterns in transmission intensity [20], antibody responses are long-lived and, unless sampling is restricted to very young age groups, additional tools are required to quantify on-going transmission. The polymerase chain reaction (PCR) is a highly sensitive method for detecting *Plasmodium* infection at all levels of endemicity [21-23]. In a meta-analysis comprising 106 surveys, microscopy detected 54.1% of all PCR-detected infections; a figure that decreased to below 20% in low endemic settings [24]. Sub-microscopic parasite carriage has been shown to contribute significantly to the malaria infectious reservoir [25,26] and is therefore of relevance for inclusion in control programmes. Actively identifying infected individuals using PCR may, therefore, be critically important when attempting to interrupt malaria transmission [7,27,28]. While PCR is commonly used as gold standard for detecting all parasitaemic individuals, there is variation between different PCR approaches [29,30] and DNA extraction from filter papers may vary in efficiency [30,31].

In the context of malaria elimination, there is a need to optimize molecular and serological assays for rapid and simultaneous assessment of the significant numbers of samples that will be generated by large scale, long term surveillance [32]. At present, DNA extraction and antibody elution are the most time consuming and laborious aspects of serological and molecular assessments. It would be operationally attractive to source DNA and antibodies from the same blood spots, as this would allow serology and PCR to be conducted in unison, increasing throughput while decreasing costs.

Here, a simple method for concurrently extracting antibodies and DNA from filter paper blood spots is presented. Antibody responses to malaria antigens are assessed to compare the efficacy of antibody elution. PCR assays using two different target genes are compared, and two sources of variation in PCR outcome are explored: the distribution of parasite material on filter papers and the amount of filter paper material that is used for DNA extraction.

## Methods

### Study area and subjects

Blood spot samples were collected in 2011 as part of a cross-sectional study in the Western Kenyan Highlands (latitude −0.470431°, longitude 34.842628°), an area of seasonal malaria transmission in which *P. falciparum* dominates. The objectives of the original study and details of the study area are detailed elsewhere [33]. Ethical approval was granted by the Scientific Steering Committee (SSC), the Ethical Review Committee (ERC) of the Kenya Medical Research Institute (KEMRI) Nairobi (proposal numbers SSC 2163, 2181 and 1589), the London School of Hygiene & Tropical Medicine ethics committee (#6111), and from Centers for Disease Control and Prevention (with exempt status) [33]. Blood derived from a finger prick was blotted onto Whatman no. 3 filter paper (Whatman, Maidstone, UK) and was dried overnight before storage with silica gel at −20°C. Each filter paper contained three individual blood spots of indeterminate volume. Filter papers were wetted through by blood spots completely, as described by Corran *et al.*[34]. A subset of 240 randomly selected blood spots was selected for both PCR and enzyme-linked immunosorbent assay (ELISA).

### Standard antibody elution

The full protocol of the elution and extraction steps is provided in the supporting documentation (Additional file 1). Three filter paper discs of 2.5 mm in diameter were punched from the centre of each dried blood spot. Filter paper discs were immediately placed into the wells of replicate 2.0 ml 96 deep well plates (Axygen Biosciences, CA, USA), one containing individual discs, the other pairs of discs. Each plate contained 80 samples so that the sample number in each corresponded to that of 2 ELISA plates, leaving wells free for controls. For standard elution single filter papers were incubated in 1120 μl of a 0.5% sodium azide/PBS solution [34]. Plates were sealed and placed onto a plate shaker on their side, allowing the cut filter paper discs to move freely along the length of their wells. After overnight incubation, the eluate was stored at −80°C. The final serum dilution of the eluate based on estimates of the volume of whole blood in a 2.5 mm filter paper disc was 1:400 [34].

### Combined antibody elution and DNA extraction

Filter paper discs were prepared and stored in deep well plates as for standard antibody elution. For combined DNA extraction and antibody elution (henceforth combined elution), 1120 μl of a 0.5% saponin/PBS solution was added to each well and plates were incubated overnight as for standard elution. 200 μl of the eluate, which contained all soluble elements including antibodies, was transferred to a new plate and stored at −80°C until use in ELISA. To continue with DNA extraction, the remaining saponin solution was aspirated and 1 ml of PBS washing solution was added to each well at 4°C. Plates were horizontally incubated on a shaker for one hour as above, before PBS was aspirated and discarded. 150 μl of a 6% Chelex in DNase/ RNase free water solution was added to each sample. Plates were sealed using adhesive foil mats (Axygen Biosciences, CA, USA) and incubated in a water bath for 3*10 minutes at 97°C. Between 10 minute incubations plates were briefly centrifuged in order to relieve pressure and ensure optimal DNA elution. After the last incubation plates were spun down at maximum speed for 5 minutes to allow the Chelex to settle. 120 μl of the DNA containing solution was taken and aliquoted into new plates. Samples were stored at −80°C until further analyses. To exclude the risk of cross-contamination during extraction materials were extensively tested using positive and negative controls (2.5% *Plasmodium* DNA and blank wells respectively). No cross-contamination was observed during extraction.

### AMA-1 and MSP-1_42_ ELISA

IgG antibody responses against AMA-1 (BPRC, 0.3 μg/ml coating concentration) and MSP-1_42_ (FVO, 0.2 μg/ml coating concentration) were detected as previously described [16,35]. Test sera were analysed in duplicate at 1:1,000 (MSP-1_42_) or 1:2,000 (AMA-1) in PBST/Marvel milk powder (Cadbury, UK). Blank wells and a serial dilution of pooled hyper-immune sera were included in duplicate on each plate to correct for non-specific antibody reactivity and standardise responses for inter-plate variation. Seroprevalence of IgG antibodies to both antigens was determined using a mixture model as described previously [16,34]. The model was used on each population of samples, giving four separate positivity thresholds (one each for AMA-1 standard elution, AMA-1 combined elution, MSP-1_42_ standard elution, and MSP-1_42_ combined elution).

### Parasite detection by PCR

Three nested PCR assays were evaluated; an 18S PCR targeting the small ribosomal subunit of *Plasmodium falciparum* developed by Snounou *et al.*[22] and two variations of a more recent assay which targets the mitochondrial cytochrome b as described by Steenkeste *et al.*[30,36]. Because of inconsistent amplification of amplicons generated by the nest 1 (N1) primers described by Steenkeste *et al.* primers of the N1 reaction were redesigned. The 18S PCR was performed according to the original protocol except that the quantity of template used in the N1 reaction was increased from 1 μl to 5 μl. In every set of PCR conditions 5 μL template was used in the N1 reaction and 1.5 μl of product in the N2 reaction. For a more detailed overview of primer sequences, product sized and PCR cycling conditions see Additional file 1. Pooled DNA extracts from *P. falciparum* NF54 cultured in Nijmegen, the Netherlands were run on every PCR plate as a positive control, alongside a negative water control. Positive control was diluted to the extent that both N1 and N2 fragments were sufficiently amplified so that both amplicons could be visualized on gel. N1 and N2 products were mixed and 10 μl was visualized on a 0.8% agarose gel by electrophoresis in 0.5 x Tris-acetate-EDTA buffer (0.04 M Tris-acetate and 1 mM EDTA, pH 8.0). Each assay was assessed using single and double filter papers, creating a total of 6 PCR conditions for comparison.

### Distribution of parasite material on filter papers

To visualize cell material in filter paper blood spots two C57BL/6 mice were infected as previously described with a transgenic *Plasmodium berghei* strain (*Pb*GFP-Luc_con_) expressing a fusion protein of GFP and Luciferase from the eef1a promoter [37,38]. The original studies that were used as a source of blood material were performed according to the regulations of the Dutch “Animal On Experimentation act” and the European guidelines 86/609/EEG; approval was obtained from the Radboud University Experimental Animal Ethical Committee (RUDEC 2009–019). 100 μl of blood from the infected mice was collected in heparinised tubes and mixed with 3.2 μl of highly concentrated (67 mg/ml) D-luciferin (Xenogen, CA, USA) dissolved in PBS [37]. 30 μl of this mixture was pipetted onto Whatman no. 3 paper (Whatman, Maidstone, UK) in a manner closely approximating that of blood spot collection in the field. Drops were first formed on the pipette tip before contact with the paper was made, and filter papers were wetted through completely. Blood spots were left to dry for 15 minutes before luminescent imaging was performed using a Lumina Caliper (PerkinElmer, MA, USA) (5 cm FOV, medium binning factor, 1 second exposure). This process was repeated for two blood spots. A blood spot without the addition of D-luciferin was used as a negative control.

### Distribution of antibody material on filter papers

Batches of 100 μl whole human blood were mixed with fluorescent labeled anti- APC-Cy7-anti-CD4 (Biolegend, CA, USA) and/or anti-human APC-IL-2. (eBioscience, CA, USA). Blood spot preparation and imaging was performed as in the cell distribution experiments.

### Data analysis

Statistical analysis was conducted using STATA 12 (StataCorp., TX, USA) and GraphPad Prism 5.0 (GraphPad Software Inc., CA, USA). IgG responses between groups of paired data were compared by Wilcoxon signed rank test. Seroprevalence comparisons were made using Chi-square test, with a test for trend in proportions. Associations between IgG responses expressed as antibody titre were quantified by Spearman correlation coefficients, and differences between elution approaches tested by linear regression presenting 95% confidence intervals (CI). The level of agreement, kappa value and sensitivity were assessed by comparing individual PCR conditions with ‘true positivity’ that was defined as positivity in any one of the PCR assay variants. The difference in the proportion of positive samples between PCR conditions was tested by McNemar’s chi-square for paired data. To minimize the influence of possible false positive PCR results on sensitivity estimates, calculations were repeated after ‘true positivity’ was defined as a positive PCR in at least two of the PCR conditions. For the cell/antibody distribution experiments Living image 3.2 was used (PerkinElmer, MA, USA). To map the relative fluorescence/intensity in different areas of the blood spot they were overlaid with grids containing cells of 2.5 mm^2^. Grids extended to the spots edges, and cells were excluded from analysis if their area was not entirely filled with dried blood. For comparison between cells, fluorescence and luminescence values were calculated as a proportion of the highest cell value.

## Results

### Antibody responses

**Figure 1:**
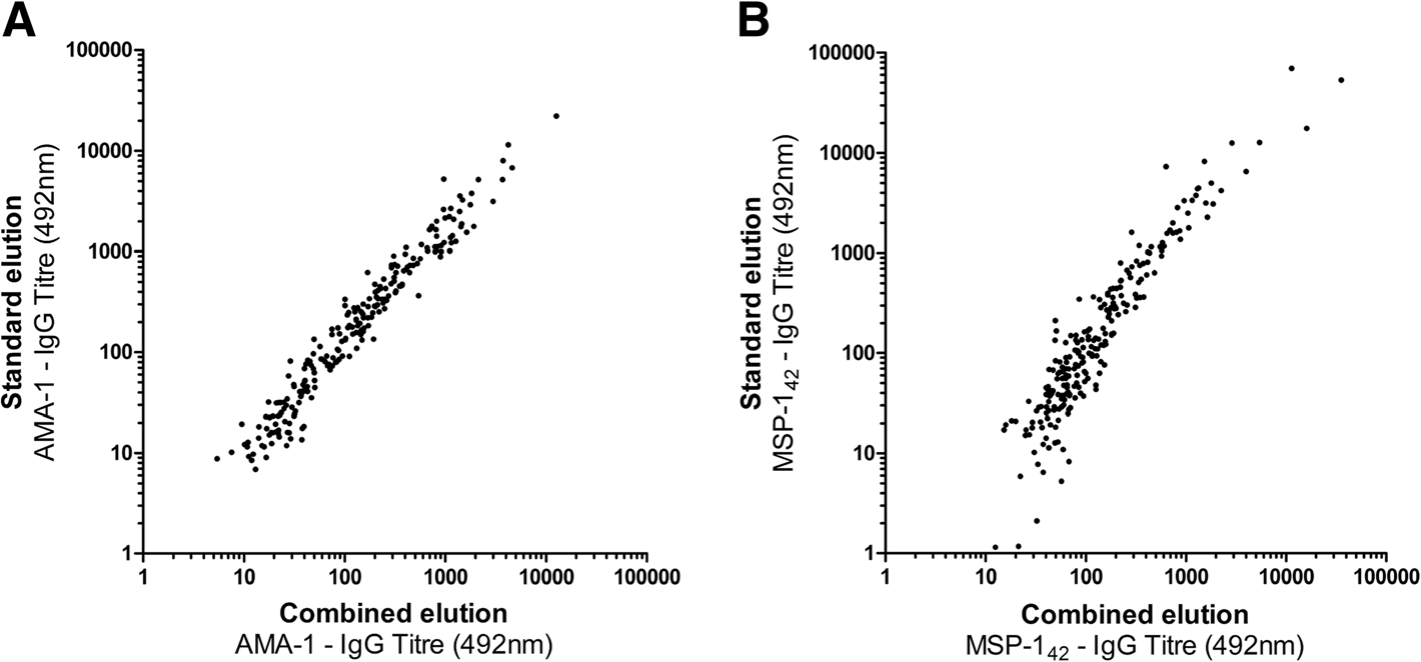
**Antibody level from standard and dual filter paper blood spot elution methods for AMA-1 and MSP-1**_**42**_**. A**. Scatter plot showing anti-AMA-1 IgG level detected in 236 individuals by standard (x-axis) and combined (y-axis) elution of filter paper blood spots. R^2^ (linear regression) = 0.93 (p = <0.0001). **B**. Scatter plot showing anti-MSP-1_42_ IgG level detected in 236 individuals using standard (x-axis) and combined (y-axis) elution of filter paper blood spots. R^2^ (linear regression) 0.671 (p = <0.0001).

**Figure 2:**
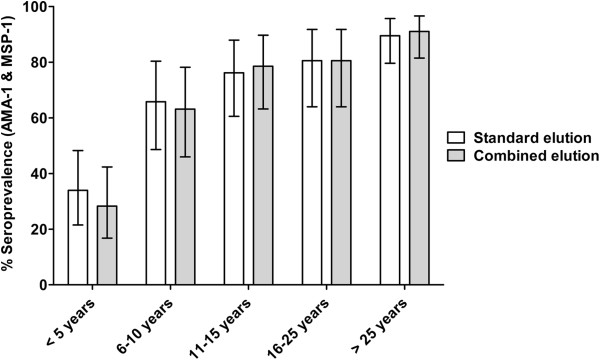
**Seroprevalence of anti-AMA-1 or MSP-1**_**42 **_**IgG responses by filter paper elution method and age.** Error bars indicate 95% confidence intervals (CI). Sample sizes for the age groups were 53 (< 5 years), 38 (6–10 years), 43 (11–15 years), 36 (16–25 years), and 67 (>25 years).

Antibodies were eluted from 236 filter papers by both the standard elution procedure and the combined elution procedure. For both AMA-1 and MSP-1_42_, a strong positive correlation was observed between the IgG responses of filter paper blood samples eluted by standard and combined methods (Figure 1). While strongly correlated, there was a tendency toward higher antibody responses when samples were eluted using the standard methodology for both antigens (p = <0.0001). For antibody level (optical density), responses were on average 11.3% higher for AMA-1, and 21.4% higher for MSP-1_42_ when using standard rather than combined elution. Linear regression analysis showed that for AMA-1 an increase of titre 1 using combined elution was associated with an increase of titre 1.773 (95% CI 1.712-1.834; p < 0.0001) using standard elution. For MSP-1_42_ an increase of titre 1 using combined elution was associated with an increase of titre 1.811 (95% CI 1.647-1.975, p < 0.0001) using standard elution. For use as marker of exposure, antibody responses against AMA-1 and MSP-1_42_ are commonly combined to give a prevalence of any anti-*P. falciparum* antibodies [17,18,20]. Between standard and combined elution methods seroprevalence of antibody responses to AMA-1 and MSP-1_42_ did not differ significantly (p >0.8), and for both methods showed a strong age-dependent increase (Figure 2; p <0.0001). Within age-groups, antibody seroprevalence did not differ significantly between elution methods (p >0.5).

### Parasite prevalence by PCR

Parasite prevalence by PCR differed between different methodologies and ranged from 12.9% (31/240) when the 18 s rRNA-based PCR was used with single filter paper discs to 19.2% (46/240) when the original cytochrome b based PCR was used with two filter paper discs (Table 1). The level of agreement between these two estimates, representing the two extremes of parasite prevalence, was high (90.4%, kappa 0.65) but the cytochrome b PCR with two filter paper discs resulted in significantly more positive results compared to the 18 s rRNA based PCR with single filter paper discs (p = 0.002). When true positivity was defined as a sample being positive in any one of the assays, the standard cytochrome b assay using DNA from two filter paper discs showed the highest sensitivity (83.6% C.I. 71.2-92.2%). Defining true positivity as a positive signal in at least two of the PCR assays did not change the estimates of sensitivity and kappa considerably (see Additional file 2).

**Table 1:** Agreement between 18 s, modified cytochrome b and original cytochrome PCR assays

**PCR assay**	**Filter paper number**	**Positivity, % (n/N)**	**Agreement, %**	**Kappa**	**Sensitivity, % (95% CI)**
18S rRNA	Single	12.9 (31/240)	90.0	0.666	56.4 (42.3 - 69.7)
	Double	16.7 (40/240)	93.8	0.804	72.7 (59.0 - 83.9)
Modified Cytochrome B	Single	15.4 (37/240)	92.5	0.760	67.3 (53.3 - 79.3)
	Double	18.3 (44/240)	95.4	0.861	80.0 (67.0 - 89.6)
Original Cytochrome B	Single	17.9 (43/240)	95.0	0.847	78.2 (65.0 - 88.2)
	Double	19.2 (46/240)	96.3	0.887	83.6 (71.2 - 92.2)

The agreement, kappa value and sensitivity were calculated by comparing individual PCR conditions with ‘true positivity’ that was defined as positivity in any one of the PCR assay variants. The abbreviation n/N indicates PCR positive individuals (n) as a proportion of the total sample size (N).

Doubling filter paper material for DNA extraction increased the sensitivity of PCR assays by 5.4-16.3%. When pairs of results from the same PCR assay but using DNA template from single or double filters were compared (n = 720 pairs), the latter resulted in significantly more parasite positive results (p = 0.013). 130 of the PCRs performed on DNA from double filter paper punches were PCR positive, compared to 111 of the PCRs performed on DNA from single filter paper punches. Surprisingly, 18.0% (20/111) of the samples that were positive in a PCR using template from a single filter punch were negative in the PCR performed when two punches were taken from the same filter paper.

Doubling filter paper material also appeared to increase the consistency of PCR outcomes on the same DNA material, albeit not statistically significant. When the 18 s rRNA-based PCR and both cytochrome b based PCRs were performed on material from the same extraction, inconsistent results (i.e. one or two but not all three PCR assays giving amplification) were observed for 8.3% (20/240) of the samples when material from single filter paper punches was used compared to 5.9% (14/240) of samples when filter paper material was doubled (Table 2, p = 0.36).

**Table 2:** Consistency of outcomes in different PCR assays in relation to the amount of filter paper material used for extraction

**Filter paper number**	**Never positive (%)**	**Positive in 1/3 PCR assays (%)**	**Positive in 2/3 PCR assays (%)**	**Positive in 3/3 PCR assays (%)**
Single	80.8 (194/240)	2.9 (7/240)	5.4 (13/240)	10.8 (26/240)
Double	79.6 (191/240)	1.3 (3/240)	4.6 (11/240)	14.6 (35/240)

The proportion (n/N) of positive PCR assays when aliquots of the same extracted material was used in three different PCR assays.

### Distribution of parasite material on blood spots

**Figure 3:**
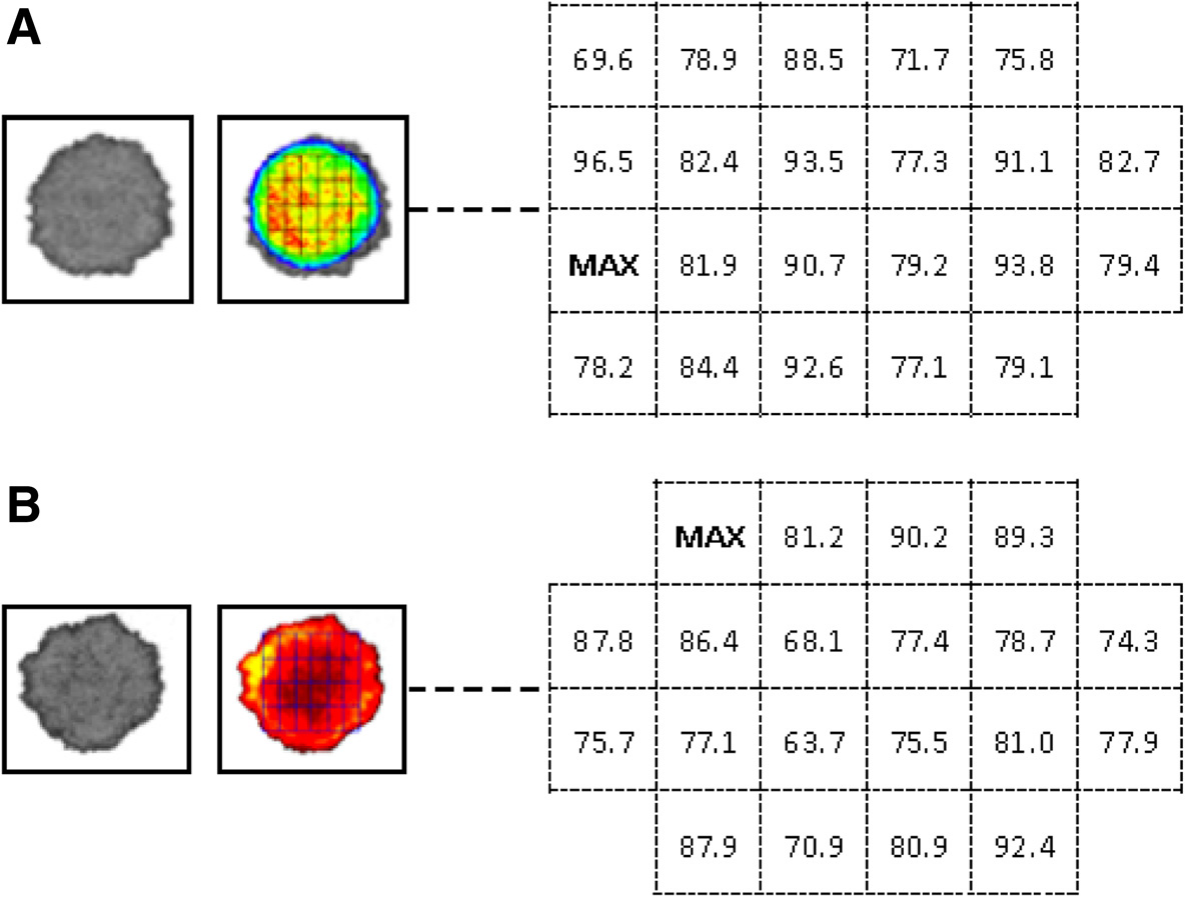
**DNA/Cell and antibody distribution on filter paper. A**. Photograph of one of the two blood spots analysed for cell material distribution, in which luminescence intensity is proportional to the density of cell material. Luminescence intensity values in the cells of the overlying grid are calculated as a proportion of the highest cell value, and are presented in the adjacent grid schema. **B**. Photograph of one of the two blood spots analysed for antibody material distribution, in which fluorescence intensity is proportional to the density of antibodies. Fluorescence intensity values are calculated and presented as in Figure 3A.

Luminescence produced by GFP expression in *Pb*-GFPluc_con_ infected blood samples was previously shown to correlate strongly with parasitaemia [39]. Here, the distribution of DNA on filter paper was assessed by measuring the luminescent intensity in dried blood spots from mice infected with *Pb*-GFPluc_con_[38]. Cell by cell luminescence analysis of the grid overlaying the blood spot was used to describe heterogeneity in parasite material in different parts of the blood spot. Both blood spots tested showed a considerable degree of heterogeneity in the distribution of parasite material (Figure 3). In the two separate experiments, 25% and 64% of the grid cells contained less than 85% of the parasite material of the grid cell with the highest quantity. Parasite material seemed less concentrated towards the extreme edges of the blood spot; the grid cell with the lowest parasite quantity contained 70% of the maximum value (Figure 3).

### Distribution of antibody material on blood spots

The distribution of antibodies on filter paper was evaluated by the adding two marker molecules which have approximately the same molecular weight of human IgG and have no detectable interaction with other components in human blood. Analysis of fluorescence intensity based on the overlaying grid showed that also antibodies were heterogeneously distributed throughout the blood spot (Figure 3). In the two separate experiments, 65% of the grid cells contained less than 85% of the parasite material of the grid cell with the highest quantity. Contrary to the observations on parasite material, there was no evident pattern in antibody concentration on the blood spot and concentrations of antibodies did not appear to be higher in the middle of the spot (Figure 3). The grid cell with the lowest antibody concentration contained 67% of the maximum value.

## Discussion

The methodology described in this report offers a cost-efficient high-throughput approach to preparing large numbers of filter paper samples for the assessment of cumulative malaria exposure and current infection status. Used in concert, serological and molecular assays can provide detailed insight into the transmission dynamics of *Plasmodium*.

The utility of serological assessments in malaria surveillance has been evidenced by numerous studies of the antibody responses of endemic populations to recombinant *Plasmodium*[40] and *Anopheles*[41-43] antigens. Recently the importance of molecular tools in malaria surveillance has been emphasised, as it has become apparent that the extent and relevance of sub-microscopic malaria infections in low endemic areas may be much greater than previously assumed [6,24]. As the number of areas making efforts to reduce or interrupt native malaria transmission grows so will the importance of sensitively detecting malaria exposure [7,32,44]. As such, the development of strategies to ease sample collection and processing during wide-scale population level surveillance is both timely and apposite to the wider malaria eradication agenda. The use of filter papers for blood collection and their subsequent storage and processing for sero-epidemiological analyses was discussed in depth by Corran *et al.*[34]. Since this time many studies have benefitted from the use of filter paper blood spots as a source of serum antibodies to reveal age-dependent [20], spatial [17-19] and temporal [45] patterns in cumulative malaria exposure. In the current study antibody levels (OD) from the standard elution methodology and the combined elution methodology (in which a portion of the filter paper eluate undergoes onward processing for DNA extraction) show a strong and highly significant correlation. Though the relationship between absolute antibody titre in paired measures was strongly related, higher antibody levels were generally observed when blood spots underwent standard elution procedures. The reason for this is unknown and may reflect differences in the relative concentration of detergent. The lower antibody yield in the combined method warns against using the two approaches simultaneously; quantitative outcomes of individual samples cannot be directly compared when different elution methods have been used. In epidemiological studies it is more common to analyse variation in malaria exposure using measures of (age-dependent) antibody seroprevalence [19,20,46]. In the current study, seroprevalence did not differ significantly between the two elution methods, and both methods showed the same age-dependent acquisition of antibody responses. This indicates that combining antibody elution with DNA extraction is an operationally attractive alternative to the standard method of antibody elution that can reliably be used to compare antibody responses between populations of blood donors.

The elution of antibodies during the process of DNA extraction adds an advantage to the chelex/saponin extraction method, which is probably the most widely used extraction method in epidemiological malaria studies. This extraction method has repeatedly been shown to give comparable results when compared to commercial extraction kits [47,48]; although in case of older or incorrectly dried and stored filter papers commercial kits may be recommended [31]. Because of its evident superior sensitivity compared to microscopy [6,24], PCR may be considered to be the gold standard for the detection of malaria infections in epidemiological studies. The current study highlights three relevant caveats to this assumption. Firstly, different PCRs differ in their sensitivity to detect malaria parasite. Although a recent meta-analysis found no differences in sensitivity compared to microscopy for different nested PCR assays [24], the current study presents evidence for a higher sensitivity of PCR based on the cytochrome b target gene compared to the most widely used 18 s rRNA target gene [31,49]. This may be due to better conservation of mitochondrial material [29]. The current study shows no advantage of the newly designed primers for the cytochrome b gene in overcoming the anecdotal problems of inconsistency of PCR results with the original protocol. The shorter N1 primers amplify the same area, may give more consistent results (data not shown) and may increase primer stability during freeze/thawing cycles but did not lead to improved sensitivity for the 240 samples tested in the current study.

Secondly, the current study presents evidence that increasing the amount of filter paper material for DNA extraction results in an increased sensitivity. Although this finding is intuitively correct, its actual relevance for determining parasite prevalence in field studies has not been described in detail before. One could argue that a single copy of template material may result in successful amplification and therefore only infections with densities close to the threshold density for detection by PCR would give discordant PCR results. The current findings suggest that this is frequently the case and that doubling filter paper material can lead to parasite prevalence estimates that are up to 3.8% higher.

Thirdly, the results illustrate the stochastic nature of PCR. Although the agreement between PCR outcomes was very high, agreement was never perfect. Discordant PCR results were common, especially if single filter paper punches were used. Importantly, some PCRs performed on single filter paper punches detected parasites while the same PCR on double filter paper punches did not. This serves as a word of warning against assuming 100% sensitivity of PCR. It has been previously acknowledged that PCR assays may fail to detect all circulating parasite clones [50,51], the current study indicates that this imperfection of PCR assays may also affect parasite prevalence estimates. The exploratory experiments described in this study on the distribution of parasite material on filter papers may be relevant in this respect: the amount of parasite material can differ by more than 15% between different punches from the same bloodspot despite the blood completely wetting the paper.

## Conclusion

When the combined DNA extraction-serum elution methodology is used, robust PCR and ELISA results can be obtained. The combined approach can significantly reduce the workload in large-scale epidemiological studies and allow efficient use of blood spot material for molecular and immunological assays. The efficient use of blood spot material may allow researchers to increase the amount of filter paper material that is used for this combined extraction. This will increase PCR sensitivity and may increase robustness of parasite prevalence estimates.

## Competing interests

The authors declared that they have no competing interests.

## Authors’ contributions

AB and WS designed the experiments, carried out the molecular and immunoassays, and drafted the manuscript. AB, JS, CD, TB designed the field studies and AB, JS and VO supervised sample collection. SS, VO and EM participated in the running of molecular and immunoassays. IP and AB participated in the cell and antibody distribution experiments. LG, JS, SK, CS, CD and RS revised the manuscript. TB conceived the experiments, aided their design, and contributed to the drafting and revision of the manuscript. All authors read and approved the final manuscript.

## Supplementary Material

Additional file 1 Combined DNA extraction and antibody elution from filter papers for the assessment of transmission intensity in epidemiological studies. Click here for file

Additional file 2 Agreement between 18 s, modified cytochrome b and original cytochrome b PCR assays when a single sample is considered a true positive when positive in at least two PCR assays. Click here for file
