# Supplementary material for: Combined DNA extraction and antibody elution from filter papers for the assessment of malaria transmission intensity in epidemiological studies
Source: Malar J. 2013 Aug 2;12:272. doi: 10.1186/1475-2875-12-272 (PMC3750228; doi:10.1186/1475-2875-12-272)
Supplement: Additional file 1 — Combined DNA extraction and antibody elution from filter papers for the assessment of transmission intensity in epidemiological studies. [file 1475-2875-12-272-S1.docx]

**Supplement 1: Combined DNA extraction and antibody elution from filter papers for the assessment of transmission intensity in epidemiological studies**

**Primer sequences and product sizes**

| **PCR assay/Nest** | **Primer name** | **Sequences 5' - 3'** | **Length (bp)** | **Amplicon size (bp)** |
| --- | --- | --- | --- | --- |
| **18S Snounou** |  |  |  |  |
| Nest 1 | rPLU6 | TTAAAATTGTTGCAGTTAAAACG | 23 | 1200 |
|  | rPLU5 | CYTGTTGTTGCCTTAAACTTC | 19 |  |
| Nest 2 | rFAL1 | TTAAACTGGTTTGGGAAAACCAAATATATT | 30 | 205 |
|  | rFAL2 | ACACAATAGACTCAATCATGACTACCCGTC | 30 |  |
| **Cytochrome B original** |  |  |  |  |
| Nest 1 | GCDW2 | CGGTCGCGTCCGGTAGCGTCTAATGCCTAGACGTATTCCTGATTATCCAG | 50 | 1253 |
|  | GCDW4 | CGCATCACCTCTGGGCCGCGTGTTTGCTTGGGAGCTGTAATCATAATGTG | 50 |  |
| **Cytochrome B modified** |  |  |  |  |
|  | CytB_1 | TTTAGCAAGTCGATATACACCAGA | 24 | 1241 |
|  | CytB_2 | CTTTAACTTGCCAACTCCCTATCA | 24 |  |
| **Cytochrome B both** |  |  |  |  |
| Nest 2 | PLAS 1 | GAGAATTATGGAGTGGATGGTG | 22 | 815 |
|  | PLAS 2 | TGGTAATTGACATCCAATCC | 20 |  |

| **Supplement 1: PCR Mastermix compositions and PCR thermal cycler programme** | | | | | |  |  |  |
| --- | --- | --- | --- | --- | --- | --- | --- | --- |
| **Snounou N1 &N2** | |  |  |  |  | **N1&N2 cycler programme** | | |
| PCR Buffer | 1x |  |  |  |  | 95°C | 5 min |  |
| MgCl2 | 2.5mM |  |  |  |  | 94°C | 60 sec | 35x |
| dNTPs | 0.2mM |  |  |  |  | 60°C | 60 sec |  |
| Primer | 0.25mM |  |  |  |  | 72°C | 90 sec |  |
| Polymerase | 2U |  |  |  |  | 72°C | 10 min |  |
|  |  |  |  |  |  |  |  |  |
| **Cytochrome B Original N1** | | | **Cytochrome B Original N2** | | | **N1&N2 cycler programme** | | |
| PCR Buffer | 1x |  | PCR Buffer | 1x |  | 95°C | 15 min |  |
| MgCl2 | 3mM |  | MgCl2 | 2mM |  | 94°C | 5 min |  |
| dNTPs | 0.2mM |  | dNTPs | 0.2mM |  | 94°C | 90 sec | 40x |
| Primer | 1µM |  | Primer | 1µM |  | 60°C | 90 sec |  |
| Polymerase | 2U |  | Polymerase | 2U |  | 72°C | 90 sec |  |
|  |  |  |  |  |  | 72°C | 10 min |  |
|  |  |  |  |  |  |  |  |  |
| **Cytochrome B new N1 & N2** | | | **N1 cycler programme** | |  | **N2 cycler programme** | | |
| PCR Buffer | 1x |  | 95°C | 5 min |  | 95°C | 5 min |  |
| MgCl2 | 2.5mM |  | 94°C | 90 sec | 40x | 94°C | 90 sec | 40x |
| dNTPs | 0.2mM |  | 62.5°C | 90 sec |  | 60°C | 90 sec |  |
| Primer | 1µM |  | 68°C | 90 sec |  | 72°C | 90 sec |  |
| Polymerase | 2U |  | 72°C | 10 min |  | 72°C | 10 min |  |

| **Supplement 1: Reagent and material list**  **Combined extraction** |  |  |
| --- | --- | --- |
| **Item** | **Supplier** | **Article code#** |
| 2 mL plate | Axygen | P-DW-20-C |
| 0.5mL plate | Corning | 3957 |
| Axygen platemax aliminium sealing film  (boiling) | Axygen | #PCR-AS-200 |
| Lids for 2mL plates | Axygen | AM-2ML-RD |
| Lids for 0.5mL plates | Thermo Scientific | AB-0674 |
| Ethanol (molecular grade) | - | - |
| Saponin | Sigma Aldrich | S4521-25G |
| Chelex 100 Resin | Bio-Rad | 143-2832 |
| PBS tablets | Sigma Aldrich | P4417-100TAB |
| H2O DNA'ase/RNA'ase free | Sigma-Aldrich | W4502-1L |
|  |  |  |
| **ELISA** |  |  |
| Immunolon plates HBX4 | Thermo Scientific | 3855 |
| Anti-Human IgG (H+L), HRP Conjugate | Promega | W4031 |
| SigmaFast OPD | Sigma-Aldrich | P9187-50SET |
| Skimmed Milk powder | Sigma-Aldrich | 70166-500G |
| TWEEN**^®^ 20** | Sigma-Aldrich | P1379-100mL |
| NaH_2_PO_4 Sodium phosphate monobasic monohydrate_ | Sigma-Aldrich | S3522-1KG |
| Na_2_HPO_4 Sodium phosphate dibasic dihydrate_ | Sigma-Aldrich | 30412-5KG |
| NaCL | Sigma-Aldrich | S7653-5KG |
| H2SO4 Sulphuric acid concentrate | Sigma-Aldrich | 32044-1EA |
|  |  |  |
| **PCR** |  |  |
| TAQ Polymerase (flexi) | Promega | M8308 |
| dNTP | Promega | U1410 |
| H2O DNA'ase/RNA'ase free | Sigma-Aldrich | W4502-1L |
| Primers | Sigma-Aldrich | - |
| Biorad flexible plates | Biorad | MLP-9601 |
| Biorad sealing film B | Biorad | MSB-1001 |
|  |  |  |

| **Standard Operating Procedure** | Version: **3**  Last revision: 12/02/13 |
| --- | --- |
| Title: Combined High - Throughput Saponin/ Chelex Extraction of DNA and Antibodies from Blood Spotted Whatman No 3 Filterpaper | |

**PURPOSE / INTRODUCTION:**

This SOP describes the detailed process of extracting and DNA and Antibodies from blood spotted Whatman No.3 filter using Saponin for lyses of cell material and elution of Antibodies and Chelex for binding and removal of oxidative elements. These filter paper samples were previously collected on filter paper and stored in the freezer at least at -20◦C. The protocol is optimized to extract material from two 2.5 millimeter discs, yielding higher amounts of DNA and Antibodies which then can be used in downstream PCR and ELISA based applications for the detection of both human and parasite DNA and parasite directed Antibodies. This protocol is fast but more prone to contamination because of the 96-well format. Therefore we recommend using the same consumables as we did.

**SHORT SUMMARY OF PROCEDURE**

Two filter paper discs of 2,5mm in diameter are taken from the center of a single dried bloodspot and added into a deep well plate incubated in 1120µL (you can use 1mL is you are only doing PCR on these samples, always use at least 1mL, adjust based on desired antibody dilution) of a 0.5% Saponin solution at RT. Occupied plates are shaken overnight. Cells will lyse and the lysate will be released into the supernatant. DNA will remain attached to a now near to clear white filterpaper disc. The saponin solution will contain serum components including antibodies. 200uL of the saponin solution is then stored in a new plate for ELISA. After a single washing step with PBS the disc(s) will be incubated in a 6% Chelex solution and finally heated at 95◦C for up to 30 minutes, while intermediately being spanned down. Chelex will bind positively charged oxidative elements leaving DNA in solution after denaturation at 95◦C. After incubation a final high speed centrifuge step follows after which the supernatant (now containing the eluted DNA) is stored in a new tube/plate with preferably no Chelex contamination.

**EQUIPMENT & CONSUMABLES**

- 2 x Tweezers (per person)
- 2x Holepunchers ( per person, 2,5 millimeter in diameter)
- Bunsen Burner
- 70% Ethanol
- Small bucket to hold Ethanol (with lid)
- Dry heat block or waterbath at 95◦C
- Racks for 10mL & 50mL tubes
- 0-200 multichannel pipette
- 200-1mL multichannel pipette
- 200uL and 1mL pipette tips
- 96 well plates (wells of 2mL+ volume) and according lids (axygen, see additional file 1)
- 96 well plates 1.2mL + lids
- Axygen sealing lids (for boiling)
- 1L/500mL Bottles

**REAGENTS**

- 100% ethanol (small bucket 50 mL for sterilization)
- 0,5% high grade saponin in PBS prepared fresh or stored at 4°C or -20°C (Sigma-Aldrich, Product Number S4521), best to have Saponin solution is at RT before use. (100mL per plate, for 1L mix add 5 grams of Saponin)
- 1X Phosphate Buffered Saline (PBS) Calcium and Magnesium free pH 7.4, stored at 4°C (100mL per plate)
- 6% Chelex-100 (Bio-rad) in DNA’ase/RNA’ase free water stored at room temperature (15mL per plate)
- Distilled water DNA/RNA’ase free

**7.0 DETAILED METHODOLOGY**

1. Organizing the plate
2. This protocol has been designed for general DNA isolation and detection of specific antibodies AMA/MSP, always check that your final saponin dilution works with your ELISA protocol.
3. For initial use, sterilize the hole puncher and forceps by flaming them in a bunsen generated flame. Dip them in the absolute ethanol and flame them off. During every sterilization step it’s essential that there is no residue alcohol on the tools, this will fixate the blood and greatly reduce DNA yield.
4. Due to the nature of our ELISA assays and the inclusion of controls you can only add 80 samples per 96-well plate leaving the last two columns reserved for controls.
5. Use the hole puncher to punch 2 X 2,5mm holes from well covered areas of the blood spot; ideally one from the center and one away from the center. Add the discs in the designated well by making use of forceps. Make sure the filter paper discs have room to move freely in the well.
6. Sterilize the hole puncher and forceps
7. Continue with the next sample in B1, fill up the plate with 80 samples
8. Leave wells in column 11 and 12 empty (control wells)

**Extraction**

1. With a multichannel pipette, add 1120uL (this will elute your serum into 1/400, check if this is correct for your own antigen) of a 0.5% Saponin solution to every well.
2. Seal every plate with a rubber sealing pad, make sure it is tightly sealed
3. Put plate on a shaker, use and intermediate shaking speed, make sure all the discs are freely moving in the plate and that the plate is properly stabilized. Leave it shaking overnight.
4. Centrifuge plate for 1 minute at high speed. Confirm that your filterpaper discs are white. If not this protocol might not yield the best quality of DNA. This is usually a result of bad storage conditions. Commercial kits might offer a better solution.
5. Transfer 200uL of the now reddish saponin to a new plate coded. This plate can either be transferred to the ELISA room for direct processing or can be stored in a freezer at -20/-80◦C for subsequent use.
6. Aspirate the rest of the saponin from the wells using a vacuum system. Try to avoid touching the filter paper discs. Preferably change tip after every well, alternatively rinse tip in PBS, if filter paper has been ‘touched’ by default replace tip.
7. Add 1 mL of cooled (+4) PBS to each tube (no saponin), seal the plate and shake for 30 minutes, again assuring that punches move freely in the well. Incubate at 4°C for 30 minutes.
8. Again shake plates for 10 minutes
9. Ensure that filter discs are properly clean.
10. As above, centrifuge and aspirate as much fluid as possible.
11. Add and adequate amount of 6% Chelex into a fluid tray (for 1 plate ~15mL). Transfer 150uL of the solution to each well using a multichannel pipette. Make sure that the Chelex is properly distributed after every transfer round. Chelex settles quickly, this step ensures that not just water is being transferred.
12. Extract the parasite DNA by incubating tubes for 3x10 minutes (a minimum of 10 minutes) in a >95°C heat-block or water bad. Every 10 minutes, spin down the plates to make sure the filter papers are still in touch with the chelex solution. To release pressure build up under the lids, either punch holes in the lid, or use tight sealing aluminum foil (Axygen).
13. After incubation, centrifuge the plates at maximum speed for 5 minutes. Prepare a new plate for storage of the DNA.
14. Transfer 120uL of the eluted DNA solution from the spun plates Make sure that the Chelex pellet is not disturbed.
15. Spin plates for 10 minutes at high speed and store at -20 or lower degrees Celsius until downstream use.

**General notes**

- Check the amount of saponin solution you have to add to reach the required antibody dilution
- It’s of essence that Chelex beads are restricted from the final PCR mixture. Since Chelex binds magnesium and other polar elements it will inhibit enzyme activity, including Taq polymerase.
- One way of doing this is by centrifuging the Chelex to a compact pellet, but this is only possible if a high speed centrifuge is present on site. Make sure that the filter was pressed to the bottom after centrifuging, so you can yield the maximum amount of clean supernatant.
- Before removing template from the final DNA plates make sure they are centrifuged.

| **Standard Operating Procedure** | Effective Date: 12/04/13 |
| --- | --- |
| High Throughput PCR detection of *Plasmodium falciparum* | |

1. **PURPOSE / INTRODUCTION:** This SOP describes the detailed process of detection of *Plasmodium falciparum* based on the detection of specific mitochondrial Cytochome B and Ribosomal 18S sequences. All assays consist of a nested PCR and conventional visualization on a agarose gel. The Cytochrome B assays are more sensitive than the more conventional Snounou 18S based PCR, and can detect parasite densities down to 1 parasite/µL. optionally a digestion reaction can be performed on the amplified product to make a distinction between Plasmodium sub species.

1. **SHORT SUMMARY OF PROCEDURE**

As indicated the PCR methods are fairly conventional consisting of two identical PCR reactions in which only the primers for the first and second reaction differ within the mastermix. Detection of amplified fragments takes place on a 1% agarose gel. For fragments sizes PCR conditions and primer sequences look at the beginning of this supplement. We measure product from both the Nested 1 and nested 2 reactions by pipetting product together before we visualize it on gel. The whole process takes place within a 96 well format reducing the need for additional pipetting steps and reducing the amount of error within the total assay. Because of in parallel DNA/Ab extraction DNA plates come filled up with 80 samples. For the PCR two controls are added.

1. **EQUIPMENT & CONSUMABLES**

- Thermal cyclers
- Gel Tanks
- Gel Trays
- Gel Casters
- 2.0mL Eppendorf tubes
- 96 well PCR plates (biorad MLP9601)
- Clean room (for the mixture of the Mastermix)
- DNA cabinet (for the addition of DNA)
- 1-10uL Multichannel/Single channel, for the addition of DNA+controls
- Pipettes 1-10/1-100/1-200/100-1000 for Mixing the Mastermix
- 5-50 12channel pipette for adding samples to gel
- Filtertips

**6.0 REAGENTS**

- Promega Taq Kit (u1510), contains MgCl2, green buffer, and 500units of Taq (100uL)
- Primers: GCDW2&4 for N1 Plas1 &2 for N2 🡪 Cytochrome B as described by Steenkeste et al.
- rPLU5&6 for N1 rFal 1 & 2 for N2 🡪 18S as described by Snounou et al.
- CytB_1&CytB_N2 Plas1 & Plas2🡪 Modified Cytochrome B
- DNA ladder properly diluted (10x)
- Positive control (pre tested, make sure that both the N1&N2 fragment are visible 5uL per plate)
- TBE buffer 0.5x
- Ethidium Bromide

**7.0 Preparing Mastermixes**

- Print labels 2*15 containing mastermix 1 or 2. Date and which primers the mix includes.
- Defrost all the components, make 15 tubes of MM1 and MM2
- Label tubes on top with MM1 & MM2 while components are defrosting
- Sterilize tubes, pipettes and tray in the UV-linker
- 15 tubes of PCR buffer (green)
- 8 tubes of MgCl2­ (properly mix)
- Defrost according amount of primer
- Defrost dNTP stocks and make a total stock of 25mm (each dNTP is 100mM)
- When tubes are defrosted spin them all down
- For 82 samples (incl. 3% error margin) per tube
- Make mastermix according to supplement 1 in 50mL tubes
- Distribute over single tubes
- Close tubes and attach labels
- Spin down
- Freeze at -20◦C in the clean room freezer

**When used:**

- Defrost
- Vortex lightly
- Add polymerase
- Shake gently, spin down and add to PCR plate using a multistep 1mL pipette
- Add 20uL per well (in 82 wells) using the multistep pipette (1mL)

**8.0 setting up PCR**

- Defrost DNA plates. Make sure that it is completely defrosted. Spin down for 5 minutes at high speed and store in fridge. Spinning down is done to make sure chelex is contained in the bottom of the well.
- Work in clean room from now on.
- Defrost mastermix tubes for a N1 reaction. Defrost and briefly spin down
- Add according amount of Taq (see supplement file 1) of Taq polymerase to every tube
- Invert tube. Briefly spin
- Use multistep pipette with 1mL tip to discard 20uL of mastermix to every well.
- Fill rows A-H, 1-10 and A11 (negative control) and B11 (positive control)
- Move to ‘dirty’ room
- Add 5uL of DNA to every according well. A1 DNA goes to A1 PCR etc.
- Make sure that you don’t mix DNA because of chelex contamination, also gently pipette up and down.
- Seal plate with Microseal B film.
- Spin down plates for 10 seconds.
- Start PCR reaction (for PCR cycling condition see supplement file 1).
- For the N2 reaction fill out N2 plates. Same as the N1 procedure except now you use N2 mastermix tubes.
- Transfer 1.5uL of DNA from the PCR plate N1 to newly made PCR plate 2.
- Spin down plates briefly (20seconds) while keeping them at 4 degrees in the meantime.
- Start N2 reaction.
- Finished N2 reaction can be stored for a few days in the fridge or freezer. We recommend to visualize it straight away or store at -20°C

**9.0 Running a gel and visualizing DNA product**

- Make a 0.8% Gel out of 0.5x TBE solution. Use 4, 26 well combs per gel. We have 2 gel tanks which can run simultaneously. (see TBE SOP 20x stock solution)
- Mix 2.4 grams of agarose with 300mL of 0.5x TBE.
- Heat in the microwave, and shake intermediately, careful since flask will be hot
- Cool down contents of flask under a stream of flowing cold water.
- Wait till flash is ‘handwarm’
- Add 6uL of EtBR. Take caution, ethidium bromide is carcinogenic. Use only specific dedicated tips and pipettes for this task and discard tips accordingly.
- Poor gel, avoiding air bubbles.
- Let the gel become solid.
- Move gel into gel tank and remove combs with caution.
- Place contrast sheets under the wells.
- Load gel with samples according to instruction.
- Add lids to gel tanks. Make sure that the poles are correctly connected to the gel power pack. Switch it on and run gel @ 180V for 45 minutes.
- Turn off gel power pack.
- Put gels on a tray and visualize product with an UV imager.
