# Supplementary material for: Combined DNA extraction and antibody elution from filter papers for the assessment of malaria transmission intensity in epidemiological studies
Source: Malar J. 2013 Aug 2;12:272. doi: 10.1186/1475-2875-12-272 (PMC3750228; doi:10.1186/1475-2875-12-272)
Supplement: Additional file 2 — Agreement between 18 s, modified cytochrome b and original cytochrome b PCR assays when a single sample is considered a true positive when positive in at least two PCR assays. [file 1475-2875-12-272-S2.docx]

**Additional File 2: Agreement between 18s, modified cytochrome b and original cytochrome b PCR assays when a single sample is considered a true positive when positive in at least two PCR assays.**

| **PCR Assay** | **Filter paper number** | **Positivity,**  **% (n/N)** | **Agreement, %** | **Kappa** | **Sensitivity,**  **% (95% CI)** |
| --- | --- | --- | --- | --- | --- |
| 18S rRNA | Single | 12.9 (31/240) | 91.3 | 0.6981 | 59.6 (45.1 - 73.0) |
|  | Double | 16.7 (40/240) | 94.2 | 0.8125 | 75.0 (61.1 - 86.0) |
| Modified Cytochrome B | Single | 15.4 (37/240) | 92.9 | 0.767 | 69.2 (54.9 - 81.3) |
|  | Double | 18.3 (44/240) | 96.7 | 0.896 | 84.6 (71.9 - 93.1) |
| Original Cytochrome B | Single | 17.9 (43/240) | 96.3 | 0.8821 | 82.7 (69.7 - 91.8) |
|  | Double | 19.2 (46/240) | 96.7 | 0.8975 | 86.5 (74.2 - 94.4) |

For sensitivity assessments, true positivity was determined as a sample being positive in at least two of the PCR assay variants. The abbreviation n/N indicates PCR positive individuals (n) as a proportion of the total sample size (N).
